# Detection of SNPs in the Cathepsin D Gene and Their Association with Yolk Traits in Chickens

**DOI:** 10.1371/journal.pone.0056656

**Published:** 2013-02-19

**Authors:** Qian Sheng, Dingguo Cao, Yan Zhou, Qiuxia Lei, Haixia Han, Fuwei Li, Yan Lu, Cunfang Wang

**Affiliations:** 1 Shandong Provincial Key Laboratory of Microbiological Engineering, Shandong Polytechnic University, Ji’nan, Shandong, China; 2 Institute of Poultry Science, Academy of Agricultural Sciences of Shandong Province, Ji’nan, Shandong, China; 3 Poultry Breeding Engineering Technology Center of Shandong Province, Ji’nan, Shandong, China; Goethe University, Germany

## Abstract

CTSD (Cathepsin D) is a key enzyme in yolk formation, and it primarily affects egg yolk weight and egg weight. However, recent research has mostly focused on the genomic structure of the CTSD gene and the enzyme’s role in pathology, and less is known about the enzyme’s functions in chickens. In this paper, the correlations between CTSD polymorphisms and egg quality traits were analyzed in local Shandong chicken breeds. CTSD polymorphisms were investigated by PCR-SSCP (polymerase chain reaction single strand conformation polymorphism) and sequencing analysis. Two variants were found to be associated with egg quality traits. One variant (2614T>C), located in exon 3, was novel. Another variant (5274G>T), located in intron 4, was previously referred to as rs16469410. Overall, our results indicated that CTSD would be a useful candidate gene in selection programs for improving yolk traits.

## Introduction

With rapid economic development and a gradual improvement in living standards, the consumer requirements for egg quality have become more stringent. Improvements in egg quality, especially the content of the egg yolk, have become a critical goal of layer hen breeding. Because it has become more difficult to improve egg quality traits by traditional breeding programs, advances in molecular genetics and the availability of DNA markers have driven the rapid progress of molecular marker-assisted breeding [1], [2].

An accumulation of variants in many genes may be associated with egg quality traits. For example, OCX32 (ovocalyxin-32) [3], prolactin [4], and LRP8 (low-density lipoprotein receptor-related protein 8) [5] have all been implicated in yolk traits. Some QTLs(quantitative trait locus) on chromosomes 2 and 4 have been identified as critical regions that are significantly associated with egg colour, egg and albumen weight, percent shell, body weight, and egg production in an analysis of 120 microsatellite markers [6], and five microsatellite loci (MCW0133, MCW0170, MCW0114, MCW0139, and LEI0074) were shown to be significantly associated with egg and yolk weights [7]. Recently, GWAS (genome-wide association studies ) have identified several loci with strong associations with yolk quality traits [1]. However, only a few genetic variants or molecular markers have shown strong evidence of an association with yolk quality traits, so the identification of genes associated with yolk quality requires further investigation.

CTSD is an important aspartic acid peptidase that plays an important role in lysosome-mediated cell protein degradation. Its main functions are protein degradation in cells [8], [9], [10]. Recent studies have found that CTSD has a close relationship with tumor invasion and metastasis in breast cancer, colon cancer, lung cancer, prostate cancer, bladder cancer and skin cancer [10], [11], [12], [13]. It has been reported that CTSD is an indicator of malignancy in serous ovarian carcinoma, and it is expressed more highly in serous ovarian carcinoma than in benign serous ovarian tumor [14]. However, CTSD expression is stable during sexual maturation [15]. CTSD is exceedingly abundant during the yolk-forming period in oocytes, and it is the key enzyme in the yolk formation process [9], [15], [16]. Thus, CTSD likely influences egg yolk and egg weights, but this link has not been studied directly. In this study, the relationship between SNPs(single nucleotide polymorphisms) in the CTSD gene and egg yolk traits was investigated in three types of Shandong chickens.

## Results

### SNPs Identified in CTSD

**Figure 1:**
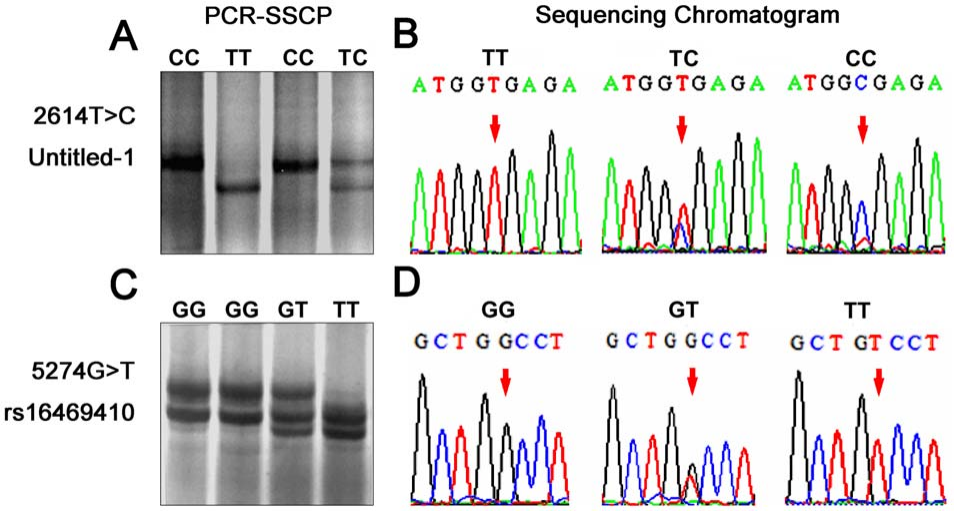
CTSD genotyping results. CTSD polymorphism genotyping with PCR-SSCP and DNA sequencing. Several samples were randomly chosen to represent the PCR-SSCP results (A and C), and DNA sequencing was used to confirm the genotyping results (B and D).

PCR-SSCP of the CTSD gene was performed, and two SNPs located at exon 3 and intron 4 were found (Figure 1). The SNP (2614T>C) located at exon 3 was a previously undiscovered variant and was named untitled 1. It was a synonymous SNP. Another variant (5274G>T), located at intron 4, was previously known as rs16469410.

The species effect was not significant; therefore, the data from the three varieties of Shandong local chicken breeds were pooled and analyzed together. As shown in Table 1, for SNP 2614T>C (untitled 1), the heterozygous TC genotype was dominant (41.9%), followed by the CC genotype and then the TT genotype, and C was an advantageous allele. For 5274G>T (rs16469410), the GG genotype was dominant (59.4%), followed by the heterozygous genotype GT and then the TT genotype, and G was an advantageous allele. In this study, the C+G haplotype was the most frequent (57.2%) (Table 2). All nine possible diplotypes for the two variants in CTSD were discovered (Table 3). The CC+GG diplotype was observed at the highest frequency (36.4%).

**Table 1:** Genotype and allele frequency of CTSD gene.

SNPs	Genotypes frequencies			Alleles frequencies	
2614T>C,untitled 1*	TT	TC	CC	C	T
	0.189(82)	0.419(182)	0.392(170)	0.601(522)	0.399(346)
5274G>T, rs16469410**	GG	GT	TT	G	T
	0.594(258)	0.339(147)	0.067(29)	0.764(663)	0.236(205)

* X^2^ = 6.82 and P value = 0.009 for Hardy-Weinberg equilibrium test,

** X^2^ = 1.63 and P value = 0.202 for Hardy-Weinberg equilibrium test.

**Table 2:** Frequencies of haplotypes based on the 2 SNPS in CTSD.

Haplotype	Observation	Frequency
C+G	497	0.572
C+T	25	0.029
T+G	166	0.192
T+T	180	0.207

**Table 3:** Least square mean of egg yolk traits in diplotypes of CTSD gene.

Diplotypes	N(freq)	Yolk weight	Yolk ratio%	Yolk diameter/mm	Yolk height/mm
CC+GG	158(0.364)	15.36±0.111B	32.34±0.152b	3.94±0.019	1.87±0.017B
CC+GT	11(0.025)	16.48±0.509A	34.60±1.049a	4.09±0.044	2.30±0.423A
CC+TT	1(0.002)	16.19±0.133AB	35.27±0.201a	4.16±0.012	1.83±0.059B
TC+GG	77(0.177)	15.85±0.128AB	32.76±0.242ab	3.90±0.037	2.00±0.049B
TC+GT	97(0.224)	15.88±0.154AB	32.88±0.213ab	3.89±0.035	1.98±0.032B
TC+TT	8(0.018)	16.06±0.377AB	33.26±0.537ab	3.94±0.096	1.94±0.112B
TT+GG	23(0.053)	15.40±0.240B	32.34±0.382b	3.90±0.061	1.90±0.051B
TT+GT	39(0.09)	15.95±0.235AB	32.78±0.362ab	3.94±0.045	1.94±0.037B
TT+TT	20(0.046)	16.11±0.194AB	32.48±0.322b	3.88±0.090	2.38±0.362A
P-value		0.0081**	0.0216*	0.5604	0.0018**

A and B mean significant at P<0.01. a, b, c and d mean significant at P<0.05 level.

* means P<0.05;** means P<0.01.

The two SNP sites were evaluated for Hardy-Weinberg equilibrium. The genotype distributions were not compatible with Hardy-Weinberg equilibrium for SNP 2614T>C (X^2^ = 6.82, P value = 0.009). However, for 5274G>T (rs16469410), the genotype distributions were in Hardy-Weinberg equilibrium (X^2^ = 1.63 and P value = 0.202).

### Associations between SNPs and Egg Yolk Traits

The correlation analysis is shown in Table 4. The 2614T>C SNP (untitled 1) was significantly associated with yolk weight (P<0.01). Additionally, 5274G>T (rs16469410) was significantly associated with yolk weight (P<0.01) and yolk height (P<0.01). The least squares mean multiple comparison of different genotype traits showed that the yolk weights of individuals with the TT and TC genotype of 2614T>C were significantly higher than those of individuals with the CC genotype (P<0.01). For SNP rs16469410, the yolk weights and yolk heights of individuals with the TT genotype were significant higher than those of individuals with the GG genotype (P<0.01).

**Table 4:** Association between two SNPs and egg yolk traits.

SNP	2614T>C, untitled-1				5274G>T, rs16469410			
Genotype	TT	TC	CC	P-value	GG	GT	TT	P-value
Yolk weight(g)	15.83±0.142A	15.88±0.099A	15.44±0.110B	0.0071**	15.51±0.082B	15.94±0.125AB	16.09±0.166A	0.0025**
Yolk ratio(%)	32.58±0.216	32.85±0.155	32.50±0.162	0.2738	32.47±0.123ab	32.98±0.190a	32.79±0.284b	0.0501*
Yolk diameter(cm)	3.91±0.035	3.90±0.025	3.95±0.018	0.2475	3.92±0.017	3.92±0.027	3.91±0.067	0.9618
Yolk height(cm)	2.03±0.092	1.99±0.027	1.90±0.031	0.0929	1.91±0.019B	1.99±0.039AB	2.24±0.252A	0.0030**

A and B mean significant at P<0.01. a, b, c and d mean significant at P<0.05 level.

* means P<0.05;

** means P<0.01.

In addition, the diplotype was significantly associated with yolk weight (P<0.01), yolk ratio (P<0.05) and yolk height (P<0.01). The least squares mean multiple comparisons of diplotype traits were shown in Table 3. The yolk weight associated with CC+GT was significantly (P<0.01) higher than that for CC+GG and TT+GG; the yolk ratios associated with CC+GT and CC+TT were significantly (P<0.05) higher than for CC+GG, TT+GG and TT+TT; and the yolk heights associated with CC+GT and TT+TT were significantly larger (P<0.01) than for the other seven diplotypes.

## Discussion

Yolk formation involves cholesterol uptake and transport mediated by the very low-density lipoprotein receptor on the membrane, and CTSD is the key enzyme in this process [17], [18]. Thus, it has been suggested that CTSD may be a candidate gene associated with egg yolk quality traits.

In the present study, the relationships between two variants of CTSD and egg yolk traits were investigated. The SNP 2614T>C, located on exon 3, is a synonymous mutation. Synonymous mutations do not change the sequence or structure of the protein, but they can change the sequence and structure of the mRNA. Thus, this SNP could affect mRNA splicing and translation. Furthermore, organismal preferences for codon usage could affect the efficiency of protein translation and further influence the adaptability of the organism [19], [20]. There is a significant relationship between gene expression and synonymous codon usage [19], [20]. For the SNP 2614T>C, the codon choice could influence CTSD protein translation, further affecting yolk formation and yolk weight. The mechanism should be studied further.

SNP 5274G>T (rs16469410), located within intron 4, also does not change the sequence or structure of the CTSD protein but is significantly correlated with egg yolk weight and egg yolk height. It was well known that many transcription factors bind sites located in the promoter or intronic regions, which could affect the efficiency of gene expressing and protein translation [21], [22]. So we hypothesize that this variant may disrupt some transcription factor-binding site, thus altering CTSD expression and affecting yolk formation. This hypothesis also needs further verification.

In conclusion, the two variants of the CTSD gene identified in this paper have highly significant effects on egg yolk weight, egg white weight, egg yolk ratio and egg yolk height. CTSD may be the major gene involved or may be linked with major genes affecting egg quality. The study of the CTSD gene could shed more light on the mechanism of egg yolk formation and provide a theoretical foundation for improving egg quality. These variants are expected to serve as useful molecular markers for assisted breeding programs.

## Materials and Methods

### Ethics Statement

The Animal Care Committee of Academy of Agricultural Sciences of Shandong Province (Ji’nan, China) approved the study. Chickens involved in the study were humanely sacrificed as necessary to reduce their suffering.

### Chickens and Egg Traits

The chicken species used for the experiment were Wenshang Luhua chickens, Laiwu black chickens and Jining Bairi chickens (150 of each) raised at the Academy of Agricultural Sciences of Shandong Province. All the three species were raised together in the same house and individual cages by staff management. Their nutrition levels were completely consistent. Venous blood samples taken from under the wing of the total 450 adult chickens were prepared for DNA extraction. Total DNA was extracted using the TIANamp Genomic DNA Kit (Tiangen, Beijing, China) according to the manufacturer’s protocol. Extracted DNA samples were stored at −20°C. Total 1350 eggs (three from each chicken) were collected for three consecutive days when the chickens were 44 weeks old. Egg yolk traits were measured daily, and the average of three eggs was used as the value for each hen. Egg yolk traits including the egg yolk weight, egg yolk height, egg yolk diameter and egg yolk ratios were determined as described in “The Poultry Production Performance Terms and Measurement Statistics Method” (NY/T823-2004).

### PCR and PCR-SSCP

CTSD gene sequence (GenBank sequence, Gene ID: 396090, NC_006092.3, GI: 358485507) were accessed from GenBank database. PCR primers were designed with the Primer Premier 5 software and shown in Table 5.

**Table 5:** Primer sequence, product sizes and annealing temperature.

Primer	Sequence of primer (5′–3′)	Length of products	Annealing temperature
CTSD- exon 3	F CATTATTGCTGGGCACCTCT	246 bp	59.2°C
	R CAGGGGCAGGATCATTATGT		
CTSD- intron 4	F GTGTTCTTCAGTAGGCTTAG	216 bp	51.4°C
	R ACTTTCACGGGACAATAA		

PCR reaction system (total volume 10 µL:2×Taq PCR MasterMix 5 µL, ddH_2_O 3.6 µL, each primer 2 µ1, DNA (50 ng/µL) 1 µL. The PCR amplification were as follows: 95°C for 5 min, followed by 35 cycles of 94°C for 30 seconds, 59.2 (51.4) °C for 30 seconds, and 72°C for 30 seconds, and ending with 6 min at 72°C. The products were visualized by electrophoresis on 12% polyacrylamide gels stained. The PCR products were purified and sequenced by Jinan Li Ge technology Co., Ltd (Jinan, China); all sequence data were analyzed with MegAlign 7.0 and Chromas 2.31 software.

### Statistical Analysis

Descriptive statistics, including tests of the normality of the distribution of traits, were calculated using univariate and means procedures with SAS software 9.1.3 (SAS Institute Inc., Cary, NC, USA). The following model was used to estimate the genetic parameters of egg yolk traits:
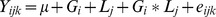
where Y = property determination value, μ = mean of the population, G = genotype effect or double type effect, L = strain effect, G*L = strain and genotype interaction effect, and e = random residual error.
